# Study on Decomposition of Indoor Air Contaminants by Pulsed Atmospheric Microplasma

**DOI:** 10.3390/s121114525

**Published:** 2012-10-29

**Authors:** Kazuo Shimizu, Tomoya Kuwabara, Marius Blajan

**Affiliations:** Organization for Innovation and Social Collaboration, Shizuoka University, 3-5-1 Johoku, Nakaku, Hamamatsu 432-8561, Japan; E-Mails: wave_the_jolly_roger@yahoo.co.jp (T.K.); blajanmarius@yahoo.com (M.B.)

**Keywords:** microplasma, dielectric barrier discharge, pulsed power, air treatment

## Abstract

Decomposition of formaldehyde (HCHO) by a microplasma reactor in order to improve Indoor Air Quality (IAQ) was achieved. HCHO was removed from air using one pass through reactor treatment (5 L/min). From an initial concentration of HCHO of 0.7 ppm about 96% was removed in one pass treatment using a discharge power of 0.3 W provided by a high voltage amplifier and a Marx Generator with MOSFET switches as pulsed power supplies. Moreover microplasma driven by the Marx Generator did not generate NOx as detected by a chemiluminescence NOx analyzer. In the case of large volume treatment the removal ratio of HCHO (initial concentration: 0.5 ppm) after 60 minutes was 51% at 1.2 kV when using HV amplifier considering also a 41% natural decay ratio of HCHO. The removal ratio was 54% at 1.2 kV when a Marx Generator energized the electrodes with a 44% natural decay ratio after 60 minutes of treatment.

## 1. Introduction

Volatile Organic Compounds (VOCs) are a problem in indoor spaces as their presence worsens the indoor air quality (IAQ). Together with malodorous substances, fungus, bacteria and tobacco smoke they cause so called sick building syndrome [1,2]. Formaldehyde (HCHO) is one of these VOCs. Its concentration in indoor air is regulated at 0.08 ppm by the Japanese Ministry of Health, Labor and Welfare (MHLW).

Active solutions can be applied for improving the indoor air quality. One of them could be an atmospheric pressure non-thermal plasma. Its generation starts from 8 kV, thus bulky power supplies and reactor are necessary [3–6]. Microplasmas, which are a dielectric barrier discharge non-thermal plasma, are generated at about 1 kV between electrodes faced together with a discharge gap on the order of micrometers. Thus the size of the needed reactor and power supply are decreased considerably, making this technology suitable for integration in home appliances such as air conditioners [7,8].

The decomposition of formaldehyde using dielectric barrier discharge was reported by Storch and Kushner [9]. A similar type of barrier discharge was used having a discharge gap of 2 mm and electrodes were energized with voltages up to 40 kV. The capability of a microplasma to remove low concentrations of HCHO in room air (below 1 ppm) for improving IAQ was investigated. The HCHO removal ratio was 97% at the discharge power of 0.3 W and 5 L/min gas flow rate. Various radicals (N*, O*, OH*, *etc.*) generated by the microplasma reacted with the VOC and decomposed it. Aroma sensory assessment was carried out by a fragrance analyzer with 10 semiconductor sensors. The microplasma changed the gas smell tendency and the odor index was reduced.

This paper presents the results of HCHO treatment by a microplasma at gas flow rates of 5 L/min. Experiments were carried out with microplasma electrodes energized by an AC and a pulse power supply in order to determine the optimal power supply for microplasma HCHO treatment.

## 2. Experimental Section

Microplasma electrodes are perforated stainless steel plates covered with a dielectric layer of 200 μm thickness. Electrodes used in this study were of circular shape Ø 58 mm, aperture ratio 36% and placed facing each other at 100 μm distance using a spacer. Due to the small discharge gap and to the dielectric constant of the dielectric layer of about εr = 10^4^, a high intensity electric field (10^7^∼10^8^ V/m) could be obtained.

**Figure 1.**
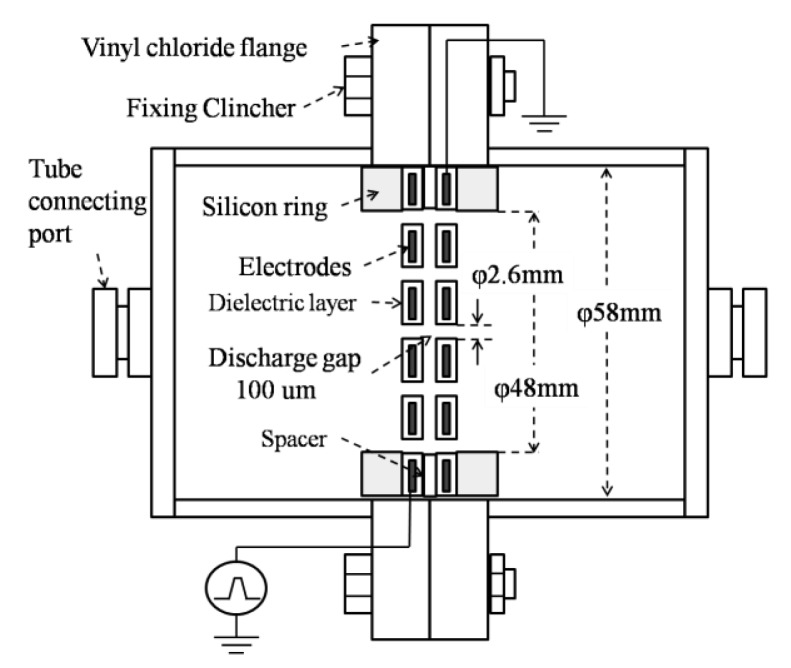
A microplasma reactor.

**Figure 2.**
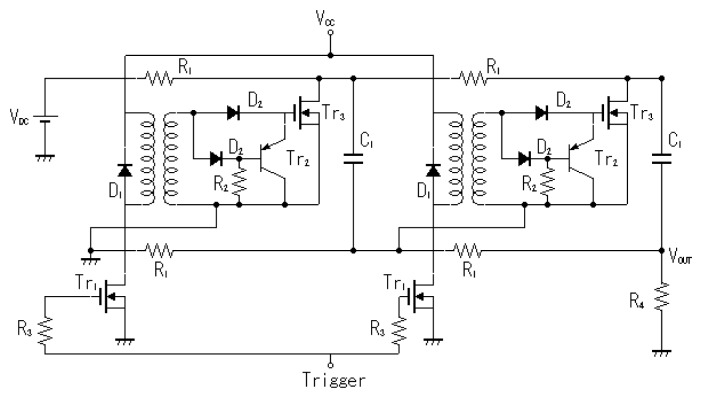
Marx generator circuit.

The microplasma reactor is shown in Figure 1. A laboratory-made Marx Generator with MOSFET switches and a pulse power supply which consisted in a function generator (Tektronix, AFG3021B) and a high voltage amplifier (Trek, MODEL 5/80) were used as power supplies to energize the electrodes. The Marx Generator generates negative pulses up to −1.6 kV, rise time 100 ns and pulse width 1.5 μs. It has two stages, as shown in Figure 2. The capacitors are linked in parallel connection when the MOSFET switches are opened. When voltage is applied the capacitors are charged at a value V and by turning on the MOSFET switches, the capacitors will discharge in a series connection multiplying the output voltage by the number of capacitors, in this case 2 V [10].

**Figure 3.**
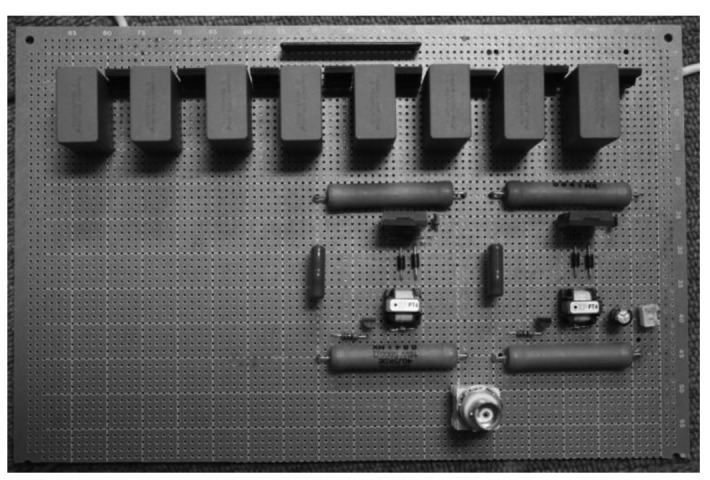
Image of the Marx Generator circuit.

The DC power supply which charges the capacitors has the maximum output of 0.8 kV, thus the Marx generator circuit can generate negative pulses up to −1.6 kV. An image of the Marx Generator circuit is shown in Figure 3. The circuit board has a relatively small size with dimensions of 160 mm × 240 mm.

**Figure 4.**
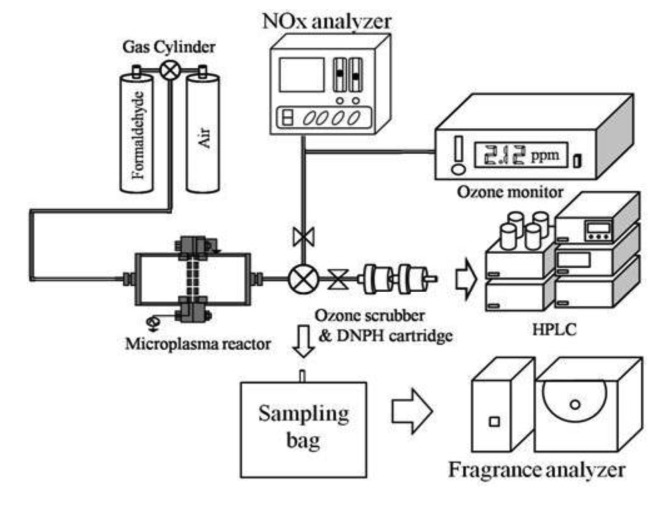
An experimental setup for one pass treatment.

Figure 4 shows an experimental setup for the one pass treatment method. A gas cylinder of HCHO (HCHO gas concentration: 19.6 ppm) and air was used to obtain a concentration of 0.7 ppm HCHO in air. Gas flow rate and gas humidity in the microplasma reactor were set at 5 L/min and 0%, respectively. The gas is flown through electrodes, thus HCHO could be decomposed due to the action of various radicals and active species (N*, N_2_*, O*, OH*, *etc.*), the intense electric field and ultraviolet light [10]. Before and after treatment the air was analyzed by an ozone monitor (Seki Electronics, SOZ-3300) for ozone quantity measurement, a NOx analyzer (Shimadzu, NOA 7000) for NOx quantity measurement, and an high performance liquid chromatography (HPLC, Agilent, 1100 series). For measuring the HCHO concentration by HPLC a dinitrophenylhydrazine (DNPH) cartridge was used to evaluate the processing ability of HCHO by the microplasma. The discharge power of an HV amplifier and a Marx Generator was set to 0.3 W to energize the microplasma electrodes. Frequency of both power supplies was set at 1.0 kHz. A fragrance analyzer (Shimadzu, FF-2A) with 10 semiconductor sensors was used for the aroma sensory assessment.

## 3. Results and Discussion

**Figure 5.**
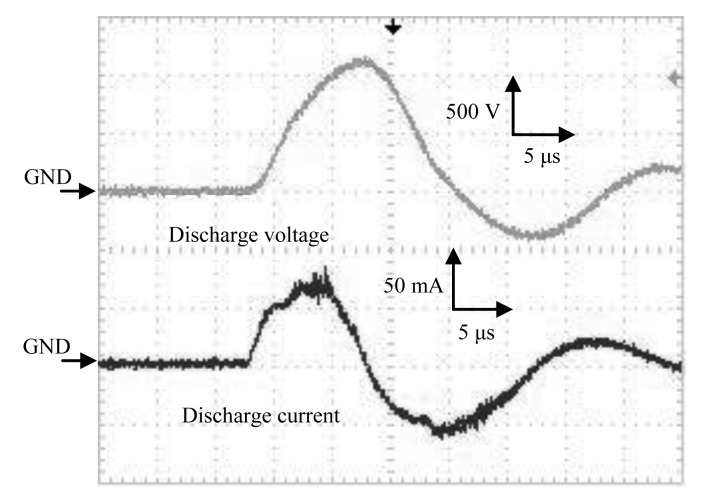
Discharge voltage and corresponding discharge current by a high voltage amplifier.

The waveforms of the discharge voltage and corresponding discharge current from the HV amplifier are shown in Figure 5. Spike currents occurred due to the microdischarges, convoluted on the current waveform, and were observed in addition to the capacitive current at steepest slopes of the waveform. Typical pulse width was about 12 μs and rising time was about 5 μs.

**Figure 6.**
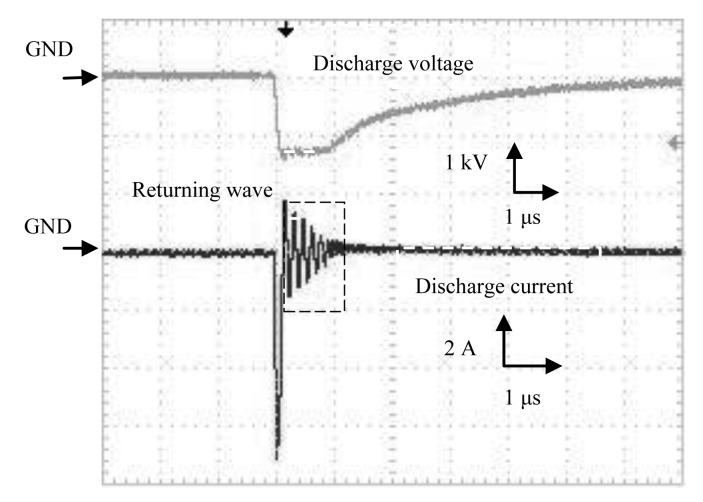
Discharge voltage and corresponding discharge current by a Marx Generator.

The waveforms of discharge voltage and corresponding discharge current from the Marx Generator are shown in Figure 6. Rise time of the discharge voltage from Marx Generator was 100 ns, and fall time was 4 μs. A sharp discharge current was observed.

**Figure 7.**
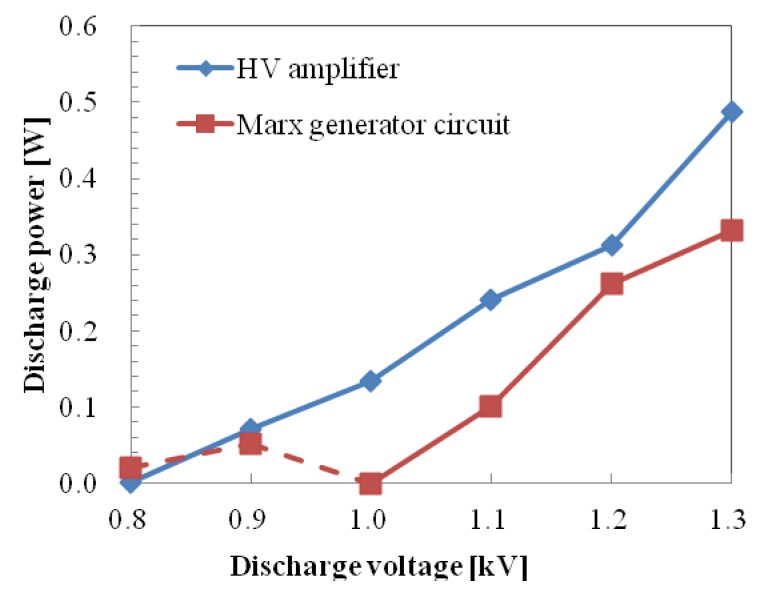
Characteristic discharge power of both power supplies.

The characteristics of discharge power *versus* discharge voltage for both the HV amplifier and Marx Generator are shown in Figure 7. Discharge power was estimated by integrating the waveform of voltage and current product in time using an oscilloscope and dividing it by time in order to obtain the power for one cycle. The current amplitude varied with the discharge voltage and had higher peak values for the Marx Generator, as shown in Figures 5 and 6.

Discharge power of the Marx Generator at 1.0 kV was lower than that at 0.9 kV. This could be explained by the decrease of transient form of the discharge current when the microplasma discharge started to occur at 1 kV. As the discharge voltage increases, the discharge power increases. Discharge power of the HV amplifier was 0.312 W at 1.2 kV for one cycle, and discharge power of the Marx Generator was 0.331 W at 1.3 kV.

As previously reported in [11], the emission spectrum from the microplasma discharge in N_2_ with water vapors when a high voltage amplifier was used to energize the electrodes, showed N_2_ Second Positive System (N_2_ SPS) peaks at 315.9, 337.1, 357.7, 367.2, 371.1, 375.5, 380.5, 394.3 nm that were observed due to electron collision [12–14], and OH peaks at 306.4 nm, 307.8 nm and 308.9 nm [15,16].

Production of OH and H radicals was due to the electron impact dissociation of H_2_O vapors [6,17]:

(1) $$\text{e}+{\text{H}}_{2}\text{O}\to \text{e}+\text{H}+\text{OH}$$

Excited state O(^1^D) could dissociate H_2_O to generate OH:

(2) O(D1)+H2O→2OHk=2.2×10−10

and also excited N_2_:

(3) $${\text{N}}_{2}*+{\text{H}}_{2}\text{O}\to \text{OH}+\text{H}+{\text{N}}_{2}\;\text{k}=4.2\times 10^{-11}$$

When air is used as carrier gas with water droplets, active species and ozone are generated by the microplasma:

(4) e+O2→e+O(P3)+O(P3,D1)

(5) $${\text{N}}_{2}({\text{A}}^{3}{\sum_{\text{u}}}^{+})+{\text{O}}_{2}\to {\text{N}}_{2}+{\text{O}}_{2}\;\text{k}=2.5\times 10^{-12}\times {\left(\text{T}/300\right)}^{0.55}$$

(6) $${\text{N}}_{2}({\text{A}}^{3}{\sum_{\text{u}}}^{+})+{\text{O}}_{2}\to {\text{N}}_{2}+\text{O}+\text{O}$$

(7) $${\text{N}}_{2}({\text{B}}^{3}\prod_{\text{g}})+{\text{O}}_{2}\to {\text{N}}_{2}+\text{O}+\text{O}\;\text{k}=3.0\times 10^{-10}$$

(8) N2(a′∑u1)−+O2→N2+O+Ok=2.8×10−11

(9) O(P3)+O2+M→O3+M

The previously mentioned active species and radicals contribute to the process of decomposition of HCHO through the following reactions:

(10) $$\text{HCHO}+\text{O}\to \text{HCO}+\text{OH}\;\text{k}=2.99\times 10^{-11}\exp(-1543/\text{T})$$

(11) $$\text{HCHO}+\text{OH}\to \text{HCO}+{\text{H}}_{2}\text{O}\;\text{k}=1.6\times 10^{-11}\exp(-110/\text{T})$$

(12) $$\text{HCHO}+\text{OH}\to \text{H}+\text{HCOOH}\;\text{k}=2\times 10^{-13}$$

(13) $$\text{HCHO}+\text{H}\to \text{HCO}+{\text{H}}_{2}\;\text{k}=3.64\times 10^{-16}{\text{T}}^{1.77}\exp(-1510/\text{T})$$

(14) $$\text{HCOOH}+\text{OH}\to {\text{H}}_{2}\text{O}+{\text{CO}}_{2}+\text{H}\;\text{k}=4.80\times 10^{-13}$$

(15) $$\text{HCO}+\text{M}\to \text{H}+\text{CO}+\text{M}\;\text{k}=8.50\times 10^{-3}{\text{T}}^{-2.14}\exp(-10278/\text{T})$$

(16) $$\text{HCO}+{\text{H}}_{2}\to \text{HCHO}+\text{H}\;\text{k}=3\times 10^{-18}{\text{T}}^{2.0}\exp(-8972/\text{T})$$

(17) $$\text{HCO}+{\text{O}}_{2}\to {\text{HO}}_{2}+\text{CO}\;\text{k}=8.50\times 10^{-11}\exp(-850/\text{T})$$

(18) $$\text{HCO}+\text{H}\to {\text{H}}_{2}+\text{CO}\;\text{k}=2\times 10^{-10}$$

(19) $$\text{HCO}+\text{O}\to {\text{CO}}_{2}+\text{H}\;\text{k}=5\times 10^{-11}$$

(20) $$\text{HCO}+\text{O}\to \text{CO}+\text{OH}\;\text{k}=5\times 10^{-11}$$

(21) $$\text{HCO}+\text{OH}\to {\text{H}}_{2}\text{O}+\text{CO}\;\text{k}=5\times 10^{-11}$$

(22) $$\text{HCO}+{\text{HO}}_{2}\to \text{OH}+\text{H}+{\text{CO}}_{2}\;\text{k}=5\times 10^{-11}$$

(23) $$\text{HCO}+{\text{H}}_{2}{\text{O}}_{2}\to {\text{CH}}_{2}\text{O}+{\text{HO}}_{2}\;\text{k}=1.70\times 10^{-13}\exp(-3486/\text{T})$$

(24) $$\text{HCO}+{\text{H}}_{2}\text{O}\to {\text{CH}}_{2}\text{O}+\text{OH}\;\text{k}=3.90\times 10^{-16}{\text{T}}^{1.35}\exp(-13146/\text{T})$$

(25) $$\text{HCO}+\text{HCO}\to {\text{CH}}_{2}\text{O}+\text{CO}\;\text{k}=3\times 10^{-11}$$

(26) $$\text{HCO}+{\text{O}}_{3}\to \text{H}+{\text{O}}_{2}+{\text{CO}}_{2}$$

The process of HCHO decomposition was reported by Storch and Kushner and they stated that the destruction of HCHO results predominantly from chemical attack by OH and O radicals via HCO. We reported in [11] that O radicals and OH radicals are the main species which are contributing to formaldehyde removal according to reactions (10) and (11). In both cases HCO results, which furthermore reacts with HCO (reaction 25) to give formaldehyde and CO or reacts with O_2_ (reaction 17) to obtain HO_2_ and CO. HO_2_ is decomposed according to reactions (27) and (28):

(27) $${\text{HO}}_{2}+\text{O}\to \text{OH}+{\text{O}}_{2}\;\text{k}=4.52\times 10^{-11}$$

(28) $${\text{HO}}_{2}+\text{OH}\to {\text{H}}_{2}\text{O}+{\text{O}}_{2}\;\text{k}=8.00\times 10^{-11}$$

**Figure 8.**
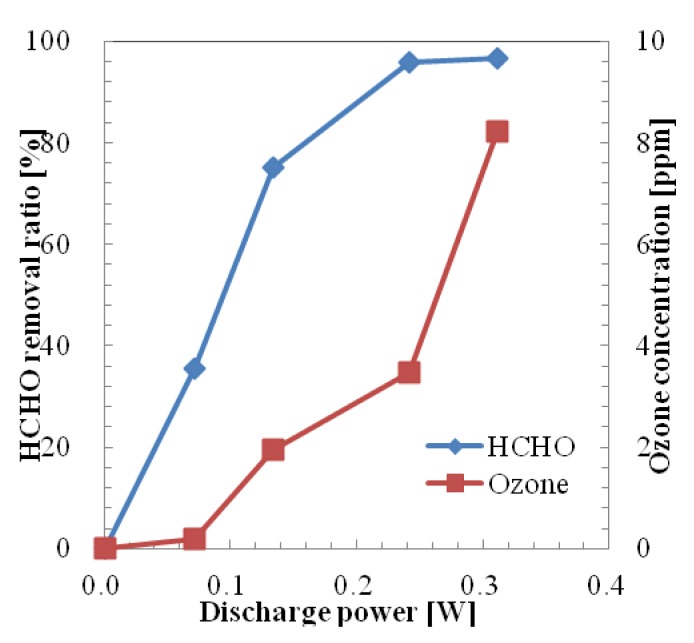
HCHO and O_3_ concentration in one pass treatment by HV amplifier.

The removal ratio of HCHO in one pass using a microplasma reactor powered by the HV amplifier by varying the discharge power up to 0.3 W is shown in Figure 8. The removal ratio was considered as (Removed Concentration × 100)/Initial Concentration. Initial HCHO concentration of the sample gas was set at 0.707 ppm by diluting HCHO (initial HCHO concentration: 19.6 ppm) by adding pure air from a gas cylinder. The removal ratio of HCHO at 0.07 W was 35.4%. At this discharge power the generated ozone concentration was quite low (below 0.2 ppm). As the value of discharge power increases the HCHO removal ratio and generated ozone concentration also increase. The HCHO removal ratio reached 95.8% at the discharge power of 0.24 W and 3.46 ppm of ozone was generated at this discharge power. The removal ratio of HCHO was saturated at around 97% when the discharge power was increased above 0.3 W. The generated ozone concentration increased with the increase of discharge power. Thus the maximum value of ozone concentration was 8.22 ppm at 0.312 W.

Ozone is known to be toxic to the human body at concentrations above 100 ppb. Thus the optimal value for the discharge voltage, in the HCHO removal process when a HV amplifier was used, could be considered 0.9 kV (discharge power: 0.07 W) due to the relatively high removal ratio of HCHO and low generated ozone concentration.

**Figure 9.**
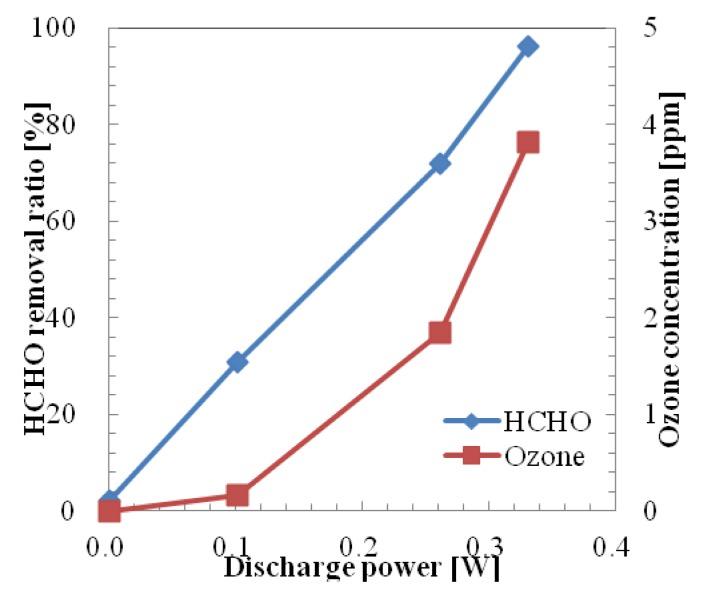
HCHO and O_3_ concentration in one pass treatment by Marx Generator.

The removal ratio of HCHO in one pass using a microplasma reactor powered by the Marx Generator is shown in Figure 9. Initial concentration of HCHO was set at 0.762 ppm. Removal ratio of HCHO was 30.7% at the discharge power of 0.102 W. At this discharge power, a low ozone concentration (0.16 ppm) was generated. The HCHO removal ratio reached 96.2% when the discharge power was increased to 0.331 W. The maximum generated ozone concentration of 3.81 ppm could be considered low, compared with the case when an HV amplifier was used.

**Figure 10.**
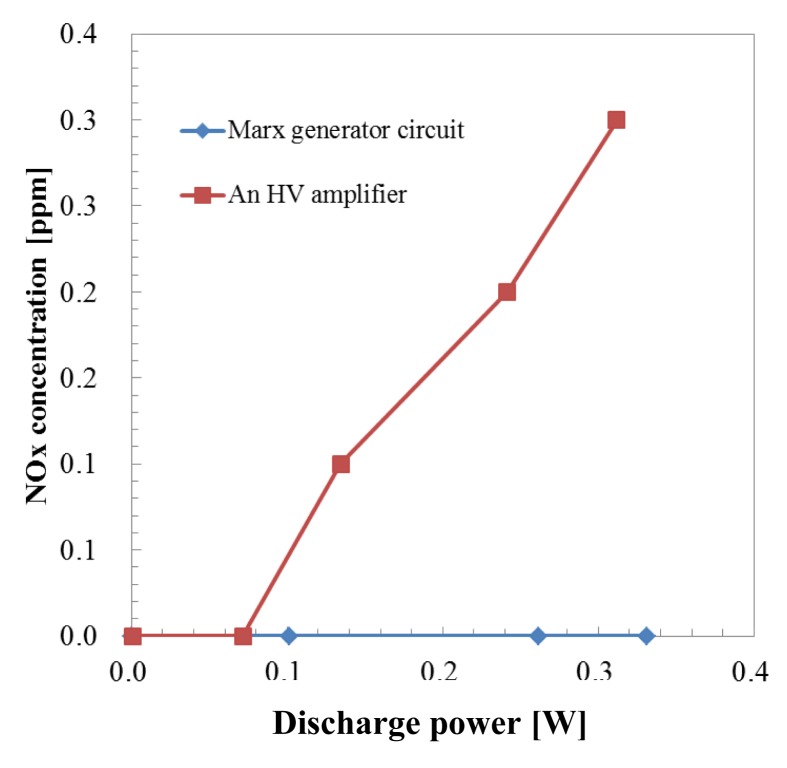
NOx generation by both power supplies.

As previously mentioned, in the one pass HCHO treatment process by microplasma the sample gas was obtained by mixing HCHO from a gas cylinder with air from another gas cylinder. Thus the humidity of the mixed gas was 0%. In the case of no humidity in air, the OH radical was not generated by the reactions (1–3). The reactions of the HCHO removal process are considered to be (10–26). NOx generation characteristics by both power supplies are shown in Figure 10.

NOx was generated according to the following chemical reactions (29–34):

(29) N2+e→N(S4)+N(S4,D2)+e

(30) $${\text{N}}_{2}+\text{e}\to {\text{N}}_{2}({\text{A}}^{3}{\sum_{\text{u}}}^{+})+\text{e}$$

(31) N2(A3∑u+)+O→NO+N∗(D2)

(32) N(D2)+O2→NO+O

(33) $${\text{O}}_{3}+\text{NO}\to {\text{NO}}_{2}+{\text{O}}_{2}$$

(34) NO+OH→NO+H

NOx started to be generated above 0.1 W when a HV amplifier was used. After increasing the discharge power more than 0.1 W, NOx concentration increased to 0.3 ppm at 0.312 W.

In the case when a Marx Generator circuit was used to generate the microplasma, no NOx generation was measured, even at 0.331 W. The discharge current of a Marx Generator circuit had short duration and generation of O_3_ was about 1/3 at same discharge voltage by an HV amplifier. According to Rousseau *et al.* NO and NO_2_ generation depends “only on the duty cycle ratio” [23]. The Duty Cycle Ratio (DCR) is defined as the ratio of the pulse duration to the pulse period. The DCRs for the Marx Generator and HV amplifier were 0.15% and 1.2%, respectively, considering the pulse width of the Marx generator and HV amplifier of 1.5 μs and 12 μs, respectively, at 1 kHz. The electron density is not constant during discharge but during the pulse on period the highest electron density corresponds to the peak of the discharge current. The DCR for the Marx Generator could be considered smaller than 0.15% due to the fact that the width of the discharge current is smaller than the width of the discharge voltage. We previously reported for the microplasma discharge in a N_2_/Ar mixture an electron density of ne = 1.6 × 10^12^ cm^−3^ calculated for the peak intensity of the discharge current [24]. Electron density was estimated also by Shao *et al.* [25] for a DBD discharge in air for a 2 mm discharge gap at 4.1 × 10^11^ cm^−3^. For an atmospheric pressure microwave-induced plasma in Ar the electron density reached ne = 3 × 10^14^ cm^−3^ [26]. This could contribute to the low NOx concentration. Considering that NOx are well known for their toxicity, in a further implementation of microplasma as a technology for cleaning the room air, the Marx Generator could be considered as the solution for the power supply.

**Figure 11.**
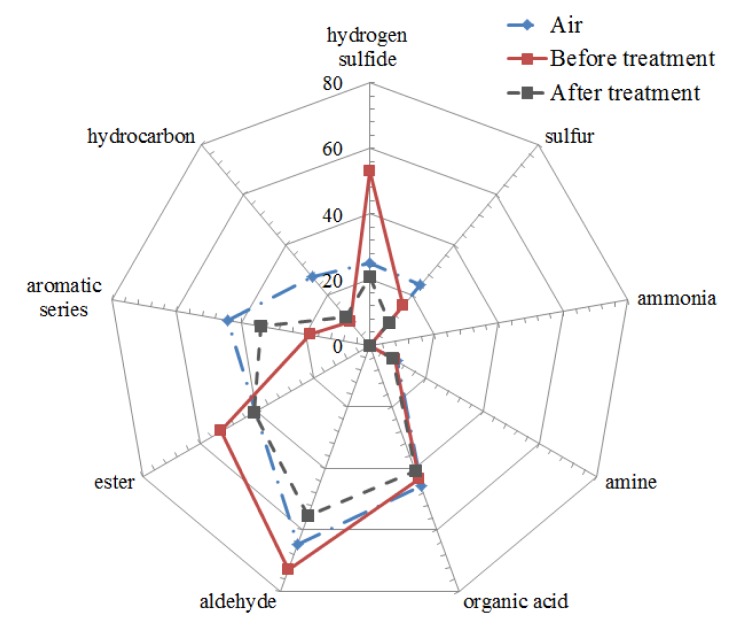
The smell similarity of before and after microplasma treatment.

**Figure 12.**
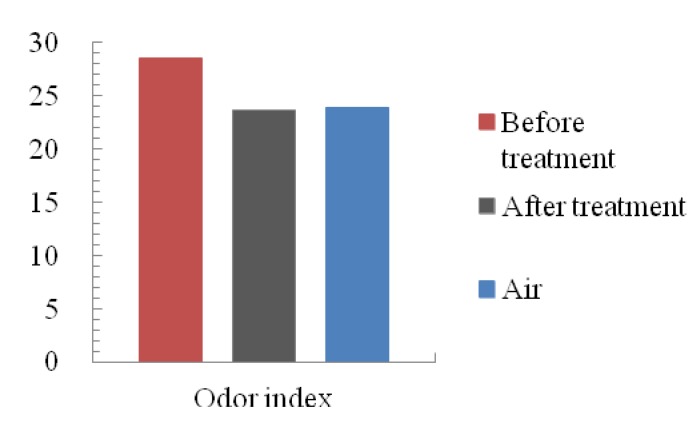
The odor index of before, after HCHO treatment and room air.

The results of smell analysis are shown in Figures 11 and 12. Ozone was collected from the sample gas by passing through an ozone scrubber (GL Science 5010-23510). The smell similarities of sample gas against various standard gases are shown as a cobweb chart in Figure 11. The highest smell similarity of sample gas before microplasma treatment was measured for the aldehyde series, next was the hydrogen sulfide series and ester series. Smell similarity of the sample gas to the aldehyde series, hydrogen sulfide and ester series was reduced after microplasma treatment. Other smell component similarity of the sample gas after microplasma treatment were also reduced or maintained at same level compared with control sample gas consisting in pure air flowed into the one pass chamber.

The value of the odor index of the control sample gas, before and after microplasma treatment gases is shown in Figure 12. Sample gas showed the highest value before treatment by microplasma. The control sample gas showed the middle value and the lowest value of odor index for the treated gas. These results show that the microplasma could also treat residual smells in the one pass chamber.

## 4. Conclusions

A high voltage amplifier and a Marx Generator were used for the removal of indoor air contaminants by microplasma. The following conclusions were obtained:

1. Removal ratio of formaldehyde using one pass microplasma reactor and a high voltage amplifier was 96.6% at the discharge power of 0.312 W with 8.22 ppm of ozone generation. The HCHO removal ratio reached 96.2% with 3.81 ppm of ozone generation at the discharge power of 0.331 W when a Marx Generator powered the one pass microplasma reactor.
2. 0.3 ppm of NOx was generated using a one pass microplasma reactor powered by an HV amplifier at a discharge power of 0.312 kV. In contrast, NOx was not observed when a Marx Generator powered the one pass microplasma reactor at any power range up to 0.331 W.
3. The similarity of bad smell components of sample gas was reduced, especially for the aldehyde series, hydrogen sulfide series and ester series after microplasma treatment. Additionally, the value of odor index was also reduced by the odor eliminating effect of the microplasma.
